# A Novel Carotenoid-Producing Bacterium, *Paenibacillus aurantius* sp. nov., Isolated from Korean Marine Environment

**DOI:** 10.3390/microorganisms11112719

**Published:** 2023-11-07

**Authors:** Chi Young Hwang, Sung Man Seo, Eui-Sang Cho, Young-Do Nam, So-Lim Park, Seong-Il Lim, Myung-Ji Seo

**Affiliations:** 1 Department of Bioengineering and Nano-Bioengineering, Incheon National University, Incheon 22012, Republic of Korea; hcyoung28@inu.ac.kr (C.Y.H.); whdmltkd123@gmail.com (E.-S.C.); 2 Advanced Geo-Materials Research Department, Pohang Branch, Korea Institute of Geoscience and Mineral Resources, Pohang 37559, Republic of Korea; smseo@kigam.re.kr; 3 Personalized Diet Research Group, Korea Food Research Institute, Wanju 55365, Republic of Korea; youngdo98@kfri.re.kr (Y.-D.N.); slpark@kfri.re.kr (S.-L.P.); 4 Division of Bioengineering, Incheon National University, Incheon 22012, Republic of Korea; 5 Research Center for Bio Materials & Process Development, Incheon National University, Incheon 22012, Republic of Korea

**Keywords:** *Paenibacillus*, marine mud, polyphasic taxonomy, isolation, carotenoid

## Abstract

The novel bacterial strain MBLB1776^T^ was isolated from marine mud in Uljin, the Republic of Korea. Cells were Gram-positive, spore-forming, non-motile, and non-flagellated rods. Growth was observed at a temperature range of 10–45 °C, pH range of 6.0–8.0, and NaCl concentrations of 0–4% (*w*/*v*). Phylogenetic analysis of the 16S rRNA gene sequence revealed that MBLB1776^T^ belonged to the genus *Paenibacillus* and was closely related to *Paenibacillus cavernae* C4-5^T^ (94.83% similarity). Anteiso-C_15:0_, iso-C_16:0_, C_16:0_, and iso-C_15:0_ were the predominant fatty acids. Menaquinone 7 was identified as the major isoprenoid quinone. The major polar lipids included diphosphatidylglycerol, phosphatidylglycerol, and phosphatidylethanolamine. Its whole genome was 6.3 Mb in size, with a G+C content of 55.8 mol%. Average nucleotide identity and in silico DNA–DNA hybridization values were below the species delineation threshold. Gene function analysis revealed the presence of a complete C_30_ carotenoid biosynthetic pathway. Intriguingly, MBLB1776^T^ harbored carotenoid pigments, imparting an orange color to whole cells. Based on this comprehensive polyphasic taxonomy, the MBLB1776^T^ strain represents a novel species within the genus *Paenibacillus*, for which the name *Paenibacillus aurantius* sp. nov is proposed. The type strain was MBLB1776^T^ (=KCTC 43279^T^ = JCM 34220^T^). This is the first report of a carotenoid-producing *Paenibacillus* sp.

## 1. Introduction

The family *Paenibacillaceae* is a well-characterized taxon within the order *Caryophanales*, with *Paenibacillus* serving as the type genus. In a previous study, the natural relationships between *Bacillus* species were elucidated and were thereby categorized into five distinct groups [1]. Among these groups, Group 3, which is closely related to *Paenibacillus polymyxa* (formerly known as *Bacillus polymyxa*), includes facultatively anaerobic species that are different from aerobic *Bacillus* strains. The inception of the genus *Paenibacillus* was proposed by Ash et al., based on the comprehensive analyses of the 16S rRNA gene and phenotypic traits [2]. Eleven *Bacillus* species were reclassified into the newly-formed genus *Paenibacillus*. Since then, the taxonomy has dynamically evolved with the continuous addition of novel *Paenibacillus* species. To date, this genus comprises 275 validly published species that span diverse habitats, including animals, insects, plants, algae, and soil.

Numerous members of *Paenibacillus* produce extracellular enzymes, polysaccharides, amino acids, and secondary metabolites [3]. Some serve as microbial inoculants that enhance plant defense through ion transport, reactive-oxygen-species-scavenging enzymes, and root hair formation [4]. However, the exploration of the pigment-producing capabilities of the genus *Paenibacillus*, including carotenoids that offer substantial biotechnological potential and commercial value, remains limited [5]. Notably, one study reported the intracellular pigment production of a *Paenibacillus* species, with erucic acid as the principal component, which has effective surfactant properties [6]. Natural pigments such as carotenoids, flavonoids, and indoles possess significant commercial value, finding applications in the development of functional foods, dyes, feed additives, and pharmaceuticals [7,8]. Particularly, the antioxidant properties of carotenoids hold promise for preventing certain human health conditions [9].

Dynamic marine ecosystems that are shaped by diverse and extreme conditions have undergone significant evolutionary changes. However, the exploration of marine organisms, especially microorganisms, is far from exhaustive. Microbes thriving in aquatic environments possess unique attributes, including halophilic, thermophilic, psychrophilic, high-pressure-adaptive, and pH-tolerant traits. Marine-derived microorganisms have gained attention because of their enzymes and metabolites that have broad applications. Marine mud, a sedimentary substrate on the seabed, hosts a considerable proportion of the microbial diversity in the biosphere [10].

In the present study, we examined the taxonomy and biological diversity of the genus *Paenibacillus* within marine mud environments. The novel strain isolated in this study sheds light on the microbial diversity in marine ecosystems. Moreover, it serves as a foundational platform for exploring microbial carotenoid production within the genus *Paenibacillus* and its promising biotechnological potential.

## 2. Materials and Methods

### 2.1. Isolation, Cultivation, and Growth Conditions

Marine mud samples were collected from the Hupo Basin in the Uljin area of the Republic of Korea (36°41′35.7″ N 129°39′29.0″ E) at a depth of approximately 200 m below the ocean surface, where muddy sediments are found. To initiate the isolation process, 0.5 g of each collected mud sample was mixed with 5 mL of 0.85% (*w*/*v*) NaCl and then vortexed. The resulting sample solutions (0.5 mL) were serially diluted in fresh Reasoner’s 2A (R2A) broth (KisanBio, Seoul, Republic of Korea). Aliquots of 100 µL from the dilution series were spread onto R2A agar plates containing 1.5% (*w*/*v*) agar. The plates were subsequently incubated at 30 °C for 2 weeks. Following incubation, colonies displaying distinct colors were chosen and sub-cultured at least three times onto fresh R2A agar plates to ensure the isolation of a pure colony. A specific orange-colored colony from the R2A agar plate was selected, designated as the MBLB1776^T^ strain, and preserved in 20% (*w*/*v*) glycerol at −80 °C. For morphological and chemotaxonomic analyses, the MBLB1776^T^ strain was cultivated on R2A agar at 30 °C for 3 d. To study its physiological characteristics and analyze pigment production, the strain was cultivated in 100 mL of R2A broth in a 250 mL baffled flask and maintained at 200 rpm. *Paenibacillus cavernae* C4-5^T^, *Paenibacillus contaminans* CKOBP-6^T^, and *Paenibacillus doosanensis* CAU 1055^T^ were purchased from the Korean Collection for Type Cultures (KCTC) and used as reference strains. This approach enabled the isolation and subsequent characterization of the novel strain MBLB1776^T^, which was further supported with comparative testing against established reference strains.

### 2.2. 16S rRNA Gene Phylogeny

The genomic DNA (gDNA) of the MBLB1776^T^ strain was extracted using a LaboPass™ Genomic DNA Isolation Kit (Cosmogenetech, Seoul, Republic of Korea). A fragment of the 16S rRNA gene was amplified with the universal bacterial primers 27F, 337F, 785F, 800R, and 1492R using polymerase chain reaction (PCR). The PCR product was purified via gel extraction using a QIAquick Gel Extraction Kit (Qiagen, Hilden, Germany), following the manufacturer’s instructions. Subsequently, the purified product was inserted into the pGEM-T Easy vector (Promega, Madison, WI, USA). The ligated plasmid was introduced into *Escherichia coli* DH5α cells and then sequenced by Macrogen Co., Ltd. (Seoul, Republic of Korea). The acquired sequences were analyzed using BioEdit v7.2.6.1 [11]. The 16S rRNA gene sequence of strain MBLB1776^T^ was deposited into GenBank (accession number: MW130723).

To elucidate the evolutionary relationship between the MBLB1776^T^ strain and closely related species, phylogenetic trees were constructed based on the 16S rRNA gene sequences using the bootstrap method with 1000 replicates. The maximum likelihood (ML), neighbor-joining (NJ), and maximum parsimony (MP) methods [12,13,14] were employed using MEGA7 [15] to quantify sequence relatedness. The Kimura two-parameter model was selected as the substitution model [16]. *Bacillus subtilis* DSM 10^T^ was used as an outgroup.

### 2.3. Phenotypic Characterization

To investigate the phenotypic traits, the MBLB1776^T^ strain was cultivated on R2A agar and broth, as previously described. Cell morphology was examined using both light (model CX 23; Olympus, Tokyo, Japan) and transmission electron microscopy (LIBRA 120; Carl Zeiss, Oberkochen, Germany). To assess cell motility, the MBLB1776^T^ strain was stabbed into 0.5% semi-solid agar tubes [17]. Gram staining was performed using a Gram stain kit (BioWORLD, Dublin, OH, USA) following the manufacturer’s guidelines. To evaluate its growth characteristics, the MBLB1776^T^ strain was cultivated to varying NaCl concentrations (0–6% *w*/*v*, increments of 1%). The optimal pH range for cell growth was determined by adjusting the pH in increments of 1.0 unit using specific buffer solutions: a 100 mM CH_3_COOH/CH_3_COONa buffer (pH 4.0–6.0), a 100 mM NaH_2_PO_4_/Na_2_HPO_4_ buffer (pH 7.0–8.0), and a 100 mM NaHCO_3_/Na_2_CO_3_ buffer (pH 9.0–10.0). The optimal temperature range for growth was assessed by incubating cells at temperatures ranging from 4 to 55 °C. Biochemical tests included the hydrolysis of casein, gelatin, starch, and Tween 20, 40, and 80 using established protocols [18]. Oxidase and catalase tests were performed using tetramethyl-p-phenylenediamine and 3% (*v*/*v*) H_2_O_2_, respectively. Anaerobic growth was assessed for 2 weeks using the GasPak^TM^ EZ anaerobic gas-generating pouch system with an indicator (BD, Franklin Lakes, NJ, USA). H_2_S production was measured using lead acetate paper and 0.5% (*w*/*v*) sodium thiosulfate. For antibiotic susceptibility testing, the following antibiotics were used (µg per disc): ampicillin (10 µg), anisomycin (50 µg), cephalothin (30 µg), erythromycin (25 µg), gentamicin (30 µg), kanamycin (30 µg), lincomycin (15 µg), neomycin (30 µg), norfloxacin (20 µg), novobiocin (10 µg), penicillin G (20 IU), streptomycin (50 µg), and tetracycline (30 µg), as mentioned in a previous publication [19]. Additional biochemical properties were explored using the API 20NE, API^®^ ZYM, and API 50 CH test kits, following the manufacturer’s instructions (bioMérieux, Marcy-l’Étoile, France).

### 2.4. Chemotaxonomic Characterization

To elucidate the chemotaxonomic properties of the MBLB1776^T^ strain, cellular fatty acids were meticulously analyzed using an Agilent 6890 gas chromatography system (Agilent, Santa Clara, CA, USA) and a cross-linked methyl siloxane column (HP-1; A30 m × 0.320 mm × 0.25 µm), as described previously [20]. This process includes cell saponification and methylation, which can extract cellular fatty acids [21]. For precise fatty acid identification and quantification, the Sherlock MIS 6.2 software and data from the TSBA6 database were used.

In addition, a YL9100 high-performance liquid chromatography (HPLC) system (Younglin, Anyang, Republic of Korea) was used to extract isoprenoid quinones. Briefly, freeze-dried cells were subjected to an established procedure for isoprenoid quinone analysis [22]. Polar lipid profiles were identified using two-dimensional thin-layer chromatography (TLC) on 10 × 10 cm silica gel 60 F254 plates (Merck, Rahway, NJ, USA) [23]. Polar lipids were visually detected by adding molybdophosphoric acid, Zinzadze’s reagent, and α-naphthol reagent on freeze-dried cells of the MBLB1776^T^ strain.

### 2.5. Whole-Genome Sequencing

The gDNA of the MBLB1776^T^ strain was extracted according to the method described above and sequenced with a Pacific Biosciences RS II instrument equipped with P6-C4 chemistry. The de novo genome assembly was performed using Flye assembler 2.7, employing the default parameters within PacBio SMRT Analysis v. 2.3.0 [24]. To ensure fidelity and absence of contamination, the whole genome was meticulously assessed in accordance with the recommended minimal standards set for utilizing the prokaryote genome database [25]. The identity of the MBLB1776^T^ strain was further confirmed by cross-referencing the 16S rRNA gene sequence derived from standard Sanger sequencing with that from whole-genome sequencing, as previously described. The whole-genome sequence of the MBLB1776^T^ strain was deposited into the National Center for Biotechnology Information (NCBI) database (NCBI accession number: GCA_032268605). To ascertain the authenticity of each sequence and detect any potential contamination within the genome assembly, the ContEst16S algorithm (https://www.ezbiocloud.net/tools/contest16s, accessed on 4 March 2021) was employed [26].

### 2.6. Comparative Genomic Analysis

A comprehensive comparative genomic analysis was conducted to gain a deeper insight into the genomic landscape of the novel strain. Genomic data, including full-genome sequences and protein sequences, were acquired from the NCBI genome database (http://www.ncbi.nlm.nih.gov/genome/, accessed on 11 July 2023) for closely related species, namely, *P. contaminans* CKOBP-6^T^ (PRJNA477746), *P. elgii* SD17^T^ (PRJDB1361), *Gorillibacterium massiliense* G5^T^ (PRJEB4162), *P. piri* MS74^T^ (PRJNA526414), and *P. doosanensis* CAU 1055^T^ (PRJNA876075). To assess genomic relatedness, the ortho-average nucleotide identity (OrthoANI) between the MBLB1776^T^ strain and the listed closely related species was computed using the OAT software (version 0.93.1) [27]. In silico DNA–DNA hybridization (*is*DDH) values were determined using the Genome-to-Genome Distance Calculator (GGDC 2.1; http://ggdc.dsmz.de/distcalc2.php, accessed on 7 July 2023). The calculation employed the recommended formula based on DNA–DNA hybridization, effectively assessing the genetic similarity between the MBLB1776^T^ strain and its closely related taxa within the genus *Paenibacillus* [28,29]. To facilitate intergenomic comparisons and evaluate the relatedness of the MBLB1776^T^ strain to other members of the *Paenibacillus* genus, the Type Strain Genome Server (TYGS) (http://tygs.dsmz.de/, accessed on 7 July 2023) was employed. A phylogenomic tree was constructed to visualize the evolutionary relationships using the FastME 2.1.4 algorithm, with support provided for branches via SPR post-processing utilizing the Genome Blast Distance Phylogeny (GBDP) distances. The numbers positioned above the branches signify the pseudo-bootstrap support values calculated based on 100 replications [30].

### 2.7. Genome Annotation and Secondary Metabolite Annotation

Comprehensive in silico genome annotation was performed using the Rapid Annotations Using Subsystems Technology (RAST) server (http://rast.nmpdr.org/, accessed on 7 July 2023) [31,32]. This annotation process is instrumental in assigning functions to genes and shedding light on the potential biological roles of various genetic elements within the genome. To further dissect the genetic landscape, predicted homologous genes were categorized into gene functions of the clusters of orthologous groups (COG) categories using EggNOG v5.0 [33,34]. Secondary metabolite biosynthetic gene clusters (BGCs) within the genome were identified using antiSMASH 6.0, and stringent detection criteria were applied to ensure accuracy [35]. Various algorithms including KnownClusterBlast, ClusterBlast, SubClusterBlast, ActiveSiteFinder, and RREFinder were used to extract additional features and annotations from these clusters. To enhance the reliability of gene function assignments, the role of each gene was validated using NCBI BLAST (https://blast.ncbi.nlm.nih.gov/, accessed on 22 June 2023), corroborating the findings from the annotation process.

### 2.8. Carotenoid Extraction and HPLC Analysis

The culture of the MBLB1776^T^ strain was centrifuged at 12,000× *g* for 5 min to collect bacterial cells. Subsequently, the cells were treated with 5 mL of acetone/methanol (7:3, *v*/*v*). To facilitate the thorough decolorization of the cells, the pellet–organic solvent suspension was incubated at 4 °C and 200 rpm overnight in the dark. After ensuring proper cell decolorization, the upper organic layers of the extracts were pooled via centrifugation at 12,000× *g* for 15 min. The collected extracts were evaporated at 40 °C using a Clever Evaporator C1 (BioChromato, San Diego, CA, USA), after which they were redissolved in methanol/dichloromethane (5:5, *v*/*v*). To obtain pure carotenoid samples, the extracts were filtered using a nylon syringe filter with a pore size of 0.2 μm (GVS Korea Ltd., Namyangju, Republic of Korea). The maximum absorbance spectra of the crude carotenoid extracts were analyzed using a YL9100 Plus HPLC system equipped with a YL9160 photodiode array (PDA) detector (Youngin Chromass, Anyang, Republic of Korea). A C30 reverse-phase (RP) column (dimensions: 5 μm, 250 mm length, and 4.6 mm inner diameter; YMC Co., Kyoto, Japan) was used for separations. Each injection (20 μL) was subjected to separations using a gradient eluent composed of methanol/water (92:8, *v*/*v*) and 10 mM ammonium acetate as the solvent A. Solvent B, composed of 100% tert-butyl methyl ether, served as the mobile phase. The carotenoid extracts were eluted for 20 min, initially with a gradient of 90% solvent A and 10% solvent B. A linear gradient was then used, reaching 83% solvent A and 17% solvent B at the 29 min mark, followed by a sharp linear gradient for 35 min to reach 30% solvent A and 70% solvent B. Ultimately, a linear gradient was initiated to attain 25% solvent A and 75% solvent B at the 42 min mark. Balance was achieved by reverting to the original conditions for over 60 min. The profiles were continuously recorded using a PDA detector operating between 200 and 600 nm, maintaining a flow rate of 1 mL/min throughout the HPLC analysis [36].

## 3. Results and Discussion

### 3.1. Phylogenetic Analysis

**Figure 1:**
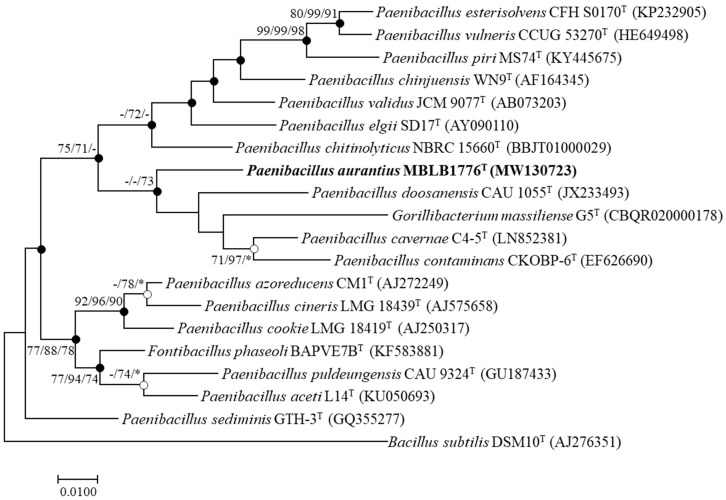
Maximum likelihood (ML) phylogenetic tree of MBLB1776^T^ strain and related type species based on the 16S rRNA gene sequences. The closed circles represent nodes recovered via both the neighbor-joining (NJ) and maximum parsimony (MP) algorithms; the open circles represent nodes recovered via either NJ or MP. The numbers on the nodes indicate the bootstrap values (>70%) and the symbol (-) indicates a value below 70% calculated using the ML/NJ/MP probabilities. The symbol (*) also indicates that it was not clustered within the algorithm. Bar, 0.01 changes per nucleotide.

A partial 16S rRNA gene sequence of the MBLB1776^T^ (1469 bp) strain was acquired using Sanger sequencing. Phylogenetic analysis using the 16S rRNA gene sequences revealed that the MBLB1776^T^ strain had a similarity below 94.8% with other closely related taxa (Supplementary Table S1). The most closely related species were *P*. *cavernae* C4-5^T^ (94.8%), *P*. *contaminans* CKOBP-6^T^ (94.1%), *P*. *elgii* SD17^T^ (93.8%), and *P*. *chinjuensis* WN9^T^ (93.7%). To construct robust phylogenetic information, the position of the MBLB1776^T^ strain was analyzed using ML, NJ, and MP trees. These analyses revealed the MBLB1776^T^ strain clustered with *P*. *cavernae* C4-5^T^, *P*. *contaminans* CKOBP-6^T^, *G*. *massiliense* G5^T^, and *P*. *doosanensis* CAU 1055^T^ (Figure 1). While acknowledging the close relationships with the mentioned species, it is worth noting that the phylogenetic tree underscores the distinction between the MBLB1776^T^ strain and most of the type strains within the genus *Paenibacillus* [37]. The MBLB1776^T^ strain occupied a distinct position within the evolutionary landscape of the genus *Paenibacillus*, indicating that it is a novel and previously undescribed species within this genus. Thus, phylogenetic analysis acts as a pivotal cornerstone in unveiling the taxonomic uniqueness of the MBLB1776^T^ strain.

### 3.2. Morphological, Physiological, and Biochemical Characteristics

Cells of the novel MBLB1776^T^ strain were Gram-positive, non-motile, spore-forming, and rod-shaped, with a width and length of 0.9–1.1 and 2.5–3.0 µm, respectively (Supplementary Figure S1). Colonies were orange, circular, and flattened, as observed on R2A plates incubated at 30 °C for 3 d. Growth was observed at 10–45 °C (optimum, 30 °C) and pH 6.0–8.0 (optimum, pH 7.0), cells could survive in a NaCl concentration of up to 4% (*w*/*v*) (optimum, 1% NaCl). The MBLB1776^T^ strain was incapable of hydrolyzing casein, gelatin, and starch, as well as Tween 20, 40, and 80. Negative reactions in the oxidase and catalase assays were observed. The MBLB1776^T^ strain did not grow under anaerobic conditions. In fact, as the deep-sea environment as the sample source for the MBLB1776^T^ strain is characterized by nearly anoxic conditions, it is reasonable to consider that the strain may be facultative anaerobic. However, it is worth noting that almost all *Paenibacillus* species are known to produce endospores, which was also confirmed in the MBLB1776^T^ strain. Therefore, it is reasonable to propose that the MBLB1776^T^ strain may exist in anaerobic deep-sea environments in the form of endospores. The MBLB1776^T^ strain did not produce H_2_S. The antibiotic susceptibility assay demonstrated that the MBLB1776^T^ strain was resistant to ampicillin, anisomycin, and penicillin G, and was susceptible to cephalothin, erythromycin, gentamicin, lincomycin, kanamycin, norfloxacin, novobiocin, neomycin, streptomycin, and tetracycline. The detailed characteristics of the MBLB1776^T^ strain and related species using the API 20NE and API^®^ ZYM kits are shown in Table 1. The API50CH profile of the MBLB1776^T^ strain is presented in Supplementary Table S2.

### 3.3. Chemotaxonomic Characteristics

The cellular fatty acid profile of strain MBLB1776^T^ showed that anteiso-C_15:0_ (56.3%), iso-C_16:0_ (10.1%), C_16:0_ (6.1%), and iso-C_15:0_ (6.0%) are the most prevalent fatty acids. These results are similar to those of the closely related type strains of the genus Paenibacillus (Supplementary Table S3). Isoprenoid quinone analysis showed that menaquinone 7 (MK-7) is the predominant isoprenoid quinone in strain MBLB1776^T^. In addition, the polar lipid profiles demonstrated that diphosphatidylglycerol (DPG), phosphatidylglycerol (PG), and phosphatidylethanolamine (PE) are the major polar lipids in strain MBLB1776^T^. Three amino polar lipids (APLs) and phospholipids were found to have minor distributions (Supplementary Figure S2).

### 3.4. Genome Information and Authenticity

The genome of the strain MBLB1776^T^ contained one complete circular chromosome. The total genome size was 6,303,770 bp, and the DNA G+C content was 55.8% (Table 2). To verify the authenticity of the genome, the 16S rRNA gene sequences obtained using whole-genome and conventional Sanger sequencing methods were compared, and the results confirmed 100% sequence similarity. The authenticity of the genome of strain MBLB1776^T^ was thus verified, as the genome sequences were not contaminated.

### 3.5. Phylogenomic Analysis

**Figure 2:**
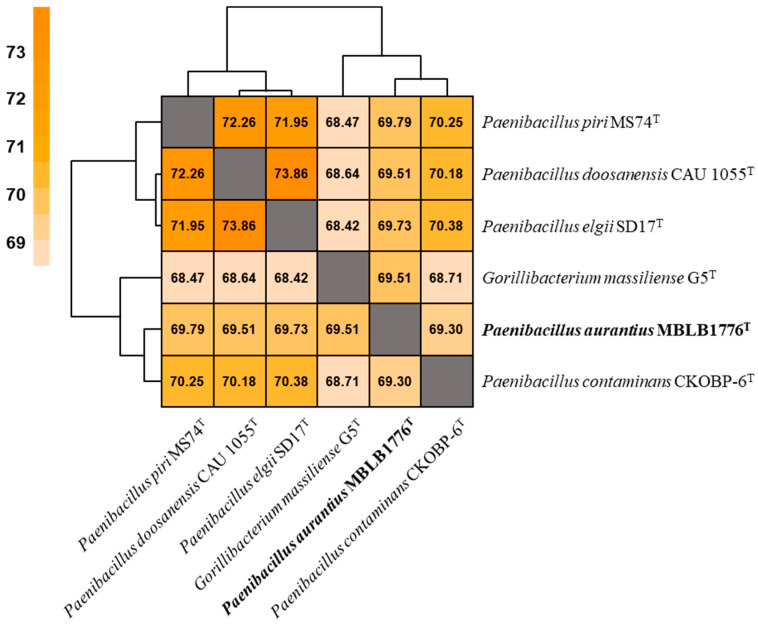
Heatmap based on OrthoANI values calculated for *Paenibacillus aurantius* MBLB1776^T^ and other closely related type strains: *P. contaminans* CKOBP-6^T^, *P. elgii* SD17^T^, *P. piri* MS74^T^, *G. massiliense* G5^T^, and *P. doosanensis* CAU 1055^T^. High OrthoANI value is indicated in deep orange, whereas lower value is indicated in pale orange.

**Figure 3:**
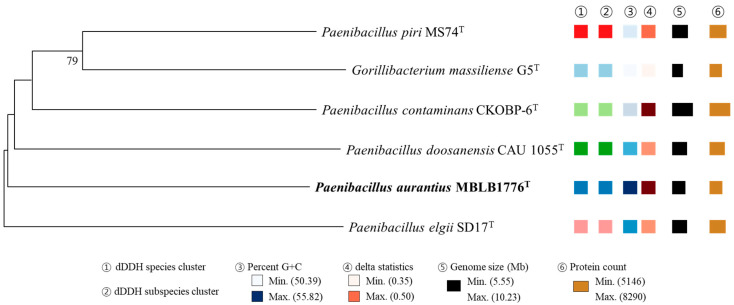
Phylogenomic tree based on TYGS results showing the relationship between strain MBLB1776^T^ with related type strains. The whole-genome sequence-based tree was generated with FastME 2.1.6.1 from GBDP distances calculated from genome sequences [27]. The different colors of the dDDH species and subspecies clusters indicate that the strains in the phylogenomic tree were different. The branch lengths are scaled in terms of GBDP distance formula d5. The numbers above branches are GBDP pseudo-bootstrap support values of >60% from 100 replications, with an average branch support of 98.7%. Genomic G + C content ranges between 50.4 and 55.8%; genome size ranges from 5.6 to 10.2 Mb; and the number of proteins coded by each genome ranges between 5146 and 8290.

The OrthoANI values between strain MBLB1776^T^ and other species of the genus Paenibacillus were below 95%, which is the species delineation threshold (Figure 2). The highest OrthoANI value was 69.8% compared with P. piri MS74^T^. In addition, the isDDH values did not exceed 25.2% (Table 3). Based on the suggested cut-off values of OrthoANI and isDDH for species delineation (less than 95% and 70%, respectively) [27,28], the calculated values suggest that MBLB1776^T^ could be distinguished from other previously reported Paenibacillus species. In addition, the phylogenomic tree showing intergenomic relatedness revealed that six species, including strain MBLB1776^T^, were not involved in equivalent species or subspecies clustering (Figure 3) [30]. These results provide further evidence of the distinctiveness of MBLB1776^T^ among other closely related species.

### 3.6. Genomic Features and Bio-Functional Potential

Among a total of 5885 coding genes, 1544 genes were annotated and included in the subsystem category distribution of the RAST server. Of these, amino acids and derivatives (254), carbohydrates (254), and protein metabolism (200); cofactors, vitamins, prosthetic groups, and pigments (130); and nucleosides and nucleotides (88) were the most abundant subsystems in strain MBLB1776^T^ (Supplementary Table S4). COG analysis showed that 5189 genes were present in strain MBLB1776^T^, and 3300 (63.6%) associated with the 21 general COG functional categories were classified as functional genes, excluding those classified as functionally unknown (S). The most abundant predicted genes in strain MBLB1776^T^ belonged to carbohydrate transport and metabolism (G; 637 [12.3%]), transcription (K; 445 [8.6%]), signal transduction mechanisms (T; 294 [5.7%]), amino acid transport and metabolism (E; 289 [5.6%]), and inorganic ion transport and metabolism (P; 231 [4.5%]) (Supplementary Table S5). 

Furthermore, the genome of strain MBLB1776^T^ had six BGCs for secondary metabolites, including the genes for ectoine, lassopeptide, LAP, and T3PKS, as well as two distinct terpene clusters (Table 4). The ectoine BGC shared 75.0% cluster similarity with Streptomyces chrysomallus ATCC 11523^T^ (GenBank: AY524544), the lassopeptide BGC shared 80.0% cluster similarity with the paeninodin BGC of Paenibacillus dendritiformis C454 (GenBank: AHKH01000064), and one terpene BGC shared 33.0% cluster similarity with the carotenoid BGC of Halobacillus halophilus DSM 2266^T^ (GeneBank: FJ040212) [41,42,43]. Overall, genome analysis and secondary metabolite annotation provided a comprehensive characterization of the genetic content of strain MBLB1776^T^, offering insights into its potential biological functions and ability to produce secondary metabolites.

Specifically, we focused on terpene BGCs, driven by the absence of reports on carotenoid biosynthesis in the *Paenibacillus* species. Several genes of the carotenoid biosynthetic pathway in strain MBLB1776^T^ were located in the cluster shown in Table 5. Strain MBLB1776^T^ harbors *crt* genes responsible for the biosynthesis and desaturation of the carotenoid backbone, including genes encoding for diapophytoene synthase (*crtM*), diapophytoene desaturase (*crtN*), 4,4′-diapophytoene desaturase (*crtNa*), 4,4′-diapophytoene-ketolase (*crtNb*), and 4,4′-diapophytoene aldehyde oxidase (*crtNc*), each of which is integral to the biosynthesis of C_30_ carotenoids (Table 5). Notably, the *crtM* and *crtN* gene families have been identified across diverse members of the family *Bacillaceae*, including the genera *Bacillus*, *Metabacillus*, *Lactobacillus*, *Cytobacillus*, and *Halobacillus*. Additional genes encoding for acyltransferases and glycosyltransferases, which are related to the modification of the carotenoid backbone, were also identified. Duc et al. [44] identified pigments in the vegetative cells and spores of the halotolerant *Bacillus* species. Khaneja et al. [45] revealed that the pigmentation of a range of bacilli was caused by carotenoids. Perez-Fons et al. [44] have shown that spore-forming *Bacillus* species produce oxygenated, glycosylated, and esterified carotenoid pigments. In the carotenoid biosynthetic pathway of strain MBLA1776^T^, carotenoid biosynthetic genes overlapped with those identified in the family *Bacillaceae*. Based on the above results, strain MBLB1776^T^ is predicted to produce putative glycosyl or acyl C_30_ carotenoids with significant bio-functional properties that can be used in the pharmaceutical, cosmetic, food additive, and feed industries. In addition, the genes encoding for carotenoid biosynthetic enzymes in strain MBLB1776^T^ can be inserted into recombinant strains for the industrial production of carotenoids.

### 3.7. Identification of Carotenoid

**Figure 4:**
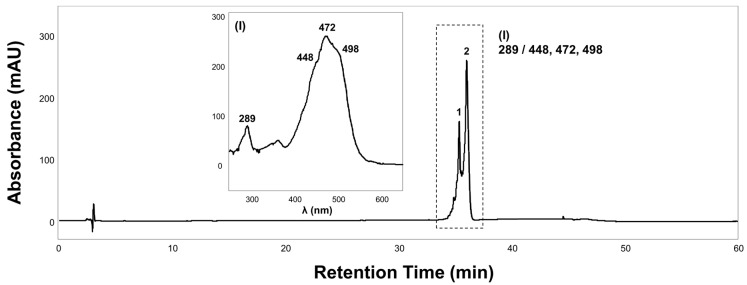
HPLC analysis of extracts from *Paenibacillus autrantius* MBLB1776^T^. Chromatogram of carotenoid extracts recorded at 472 nm and UV/Vis spectra of group I according to their chromatographic peaks.

Carotenoid extraction and subsequent HPLC analysis provided an understanding of the carotenoid content and composition in strain MBLB1776^T^. The chromatogram exhibited two primary peaks that were not fully resolved. These peaks shared a distinct signature carotenoid absorption spectrum, prominently manifesting at 472 nm with persistent occurrences at 448 and 498 nm and displayed pronounced *cis* peaks at 289 nm (Figure 4). Based on the chromatographic patterns and spectral attributes reminiscent of *Bacillus* strains previously acknowledged for their synthesis of characteristic orange carotenoids, the carotenoid synthesized by strain MBLB1776^T^ was inferred to be methyl 1-(6-C_9:0_)-glycosyl-apo-8′-lycopenoate, methyl 1-(6-C_10:0_)-glycosyl-apo-8′-lycopenoate, and methyl 1-(6-C_11:0_)-glycosyl-apo-8′-lycopenoate [44,45,46]. The spectral results for strain MBLB1776^T^ showed discrepancies with those of previously reported orange carotenoids at 438, 466, and 492 nm. These small variations may be attributed to the disparities in the molar extinction coefficients across different solvents in which the carotenoids are solubilized. Zang et al. [47] observed that the absorbance spectra of carotenoids are dependent on the solvent used, resulting in minor fluctuations in the peak absorption wavelengths. Alternatively, the *crtO* gene, which acylates C_30_ 4,4′-diapocarotenoids, may have produced a novel, previously unknown carotenoid [48]. Based on the results of the present and previous studies on this topic, we identified the tendency of carotenoid production in *Paenibacillus* species, which can provide better insights into carotenoid production in microorganisms. However, the specific carotenoids produced have not yet been extensively identified. Further analysis of the carotenoids extracted from strain MBLB1776^T^ is essential to elucidate the biosynthetic pathways and compounds involved in their biosynthesis.

## 4. Conclusions

This study presents a genome-based approach for the identification of a novel species within the genus *Paenibacillus*, the description of which is provided below. Furthermore, we suggest investigating carotenoid biosynthesis in strain MBLB1776^T^ using a genomic approach. The presence of carotenoids in the strain MBLB1776^T^ cell extract was confirmed using spectroscopic and chromatographic analyses for the first time. Via comprehensive phenotypic and genome-based investigations, we explored potential taxonomic distinctions of this strain from previously reported *Paenibacillus* species. Based on our findings, we propose that strain MBLB1776^T^ serves as the type strain for a novel species within the genus *Paenibacillus*, characterized by carotenoid production. Consequently, we introduce the designation *Paenibacillus aurantius* sp. nov.

### Description of Paenibacillus aurantius *sp. nov.*

*Paenibacillus aurantius* sp. nov. (au.ran’ti.us. N.L. masc. adj. *aurantius*, orange, pertaining to colony color).

The cells are Gram-positive, non-motile, spore-forming, and rods. The colonies are circular, flattened, and orange in color when grown on R2A agar at 30 °C for 3 d. Optimal growth is observed at a temperature range of 10–45 °C (optimum, 30 °C), pH range of 6.0–8.0 (optimum, pH 7.0), and NaCl concentrations of 0–4% (*w*/*v*) (optimum, 1% NaCl). Casein, gelatin, and starch, as well as Tween 20, 40, and 80, do not hydrolyze. They are positive for catalase, but negative for oxidase activity and H_2_S formation. Anaerobic growth is not observed. In the API 20NE test, β-glucosidase and β-galactosidase activities are positive, but the reduction of nitrate to nitrite, reduction of nitrite to nitroxide, indole production, glucose fermentation, arginine dihydrolase, urease activity, and protease (gelatin) activity are negative. In the API^®^ ZYM test, esterase lipase (C8), acid phosphatase, naphthol-AS-BI-phosphohydrolase, α-galactosidase, and β-galactosidase activities are positive, but alkaline phosphatase, esterase (C4), lipase (C14), leucine arylamidase, valine arylamidase, cystine arylamidase, trypsin, α-chymotrypsin, β-glucuronidase, α-glucosidase, β-glucosidase, N-acetyl-β-glucosaminidase, α-mannosidase, and α-fucosidase activities are negative. The major fatty acids are anteiso-C_15:0_, iso-C_16:0_, C_16:0_, and iso-C_15:0_. The predominant isoprenoid quinone is menaquinone 7 (MK-7), and the main polar lipids are DPG, PG, and PE. The genome size of the type strain is 6.3 Mb with a G+C value of 55.8 mol%. The NCBI GenBank accession number for the 16S rRNA gene sequence is MW130723, and the NCBI accession number for the whole-genome assembly is GCA_032268605. The type strain, MBLB1776^T^ (=KCTC 43279^T^ =JCM 34220^T^), was isolated from marine mud in the Uljin area of the Republic of Korea.

## Figures and Tables

**Table 1:** Differential characteristics between *Paenibacillus aurantius* MBLB1776^T^ and the type strains of closely related species in the genus *Paenibacillus* (taxa: 1. strain MBLB1776^T^; 2. *P*. *cavernae* C4-5^T^; 3. *P*. *contaminans* CKOBP-6^T^; and 4. *P*. *doosanensis* CAU 1055^T^). All of these species were negative for casein, gelatin, starch, Tween 20, 40, and 80 degradation, and H_2_S formation. All of these species gave positive results for β-glucosidase (esculin), β-galactosidase, glucose, and maltose but negative results for reduction of nitrate to nitrite, reduction of nitrite to nitroxide, indole production, glucose fermentation, arginine dihydrolase, urease, arabinose, caprate, adipate, citrate, and phenyl-acetate in the API20NE test. All of the species gave positive results for naphthol-AS-BI-phosphohydrolase and β-galactosidase but negative results for alkaline phosphate, esterase (C4), lipase (C14), valine arylamidase, cystine arylamidase, trypsin, β-glucuronidase, N-acetyl-β-glucosaminidase, α-mannosidase, and α-fucosidase in the API^®^ ZYM test.

Characteristic	1	2	3	4
Temp. range (°C) (optimum)	10–45 (30) *	20–42 (30) ^a^	10–37 (30) ^b^	4–45 (30) ^c^
NaCl range (%, *w*/*v*) (optimum)	0–4 (1) *	0–1 ^a^	0–2 (0.5) ^b^	0–4 (2) ^c^
pH range for growth (optimum)	6.0–8.0 (7.0) *	6.0–10.0 (7.0) ^a^	6.5–8.0 (7.0) ^b^	4.5–7.5 (6.5) ^c^
Catalase *	−	+	+	+
Oxidase *	−	+	+	+
API 20NE *				
Protease (gelatin)	−	+	+	−
Mannose	−	−	−	+
Mannitol	−	−	+	−
N-acetyl-glucosamine	−	+	+	−
Gluconate	+	−	+	−
Malate	+	−	+	−
API^®^ ZYM *				
Esterase lipase (C8)	+	−	+	+
Leucine arylamidase	−	−	−	+
α-chymotrypsin	−	+	−	−
Acid phosphatase	+	+	−	+
α-galactosidase	+	−	−	−
α-glucosidase	−	+	+	+
β-glucosidase	−	−	+	+

* Data from this study. ^a^ Data from [38]; ^b^ [39]; and ^c^ [40].

**Table 2:** General characteristics of the genome of *Paenibacillus aurantius* MBLB1776^T^.

Attribute	Characteristics
Sequencing platforms	PacBio
Assembler	FLYE v. 2.7
Genome coverage	111.98×
Assembly status	Complete
Assembly size (bp)	6,303,770
G + C content (mol%)	55.8
Total contigs	1
Total CDS	5588
RNAs	112
-rRNA genes (5S, 16S, 23S)	30
-tRNAs	82

**Table 3:** In silico DDH (*is*DDH) values and G + C content differences between *Paenibacillus aurantius* MBLB1776^T^ and closely related type strains.

Query Genome	Reference Genome	*is*DDH Value (%)	Model Confidence Interval (%)	G+C Content Difference (%)
MBLB1776^T^	*Paenibacillus piri* MS74^T^	25.2	[22.9–27.7]	4.78
	*Paenibacillus contaminans* CKOBP-6^T^	23.4	[21.1–25.9]	4.31
	*Paenibacillus doosanensis* CAU 1055^T^	22.7	[20.4–25.1]	2.53
	*Gorillibacterium massiliense* G5^T^	22.4	[20.1–24.8]	5.44
	*Paenibacillus elgii* SD17^T^	21.2	[18.9–23.6]	2.42

**Table 4:** Distribution of BGCs of *Paenibacillus aurantius* MBLB1776^T^ and similar known pathways with strict detection criteria.

Gene Type	Product	Span (nt)	Most Similar Biosynthetic Gene Cluster	BGC Similarity (%)
Ectoine	Ectoine	414,375–424,767	*Streptomyces chrysomallus* ATCC 11523^T^	75.0
Lassopeptide	Paeninodin	1,318,636–1,342,527	*Paenibacillus dendritiformis* C454	80.0
LAP		2,211,773–2,235,324	-	-
T3PKS		2,799,722–2,840,831	-	-
Terpene		3,482,565–3,500,973	-	-
Terpene	Carotenoid	4,537,895–4,558,767	*Halobacillus halophilus* DSM 2266^T^	33.0

**Table 5:**
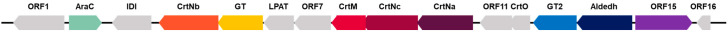
Carotenoid biosynthetic gene organization and homology analysis of *Paenibacillus aurantius* MBLB1776^T^ in terpene criteria.

Name	Top Hit	Description	E-Value	Identity (%)
ORF1	WP_138193179	Sulfatase (*Paenibacillus antri* SYSU K30003^T^)	0.0	77.2
AraC (ORF2)	WJH34801	AraC family transcriptional regulator (*Paenibacillus* sp. CC-CFT747)	0.0	98.6
IDI (ORF3)	WJH34802	Type 2 isopentenyl-diphosphate delta-isomerase (*Paenibacillus* sp. CC-CFT747)	0.0	97.7
CrtNb (ORF4)	WP_307396138	Phytoene desaturase family protein (*Bacillus horti*)	0.0	59.1
GT (ORF5)	WP_130609653	Glycosyltransferase family 2 protein (*Cohnella abietis*)	5 × 10^−147^	56.4
LPAT (ORF6)	WJH34805	Lysophospholipid acyltransferase family protein (*Paenibacillus* sp. CC-CFT747)	3 × 10^−118^	98.3
ORF7	WJH34807	Carotenoid biosynthetic protein (*Paenibacillus* sp. CC-CFT747)	4 × 10^−103^	95.3
CrtM (ORF8)	WP_189012146	Phytoene/squalene synthase family protein (*Paenibacillus marchantiophytorum* R55^T^)	3 × 10^−142^	68.5
CrtNc (ORF9)	WJH34808	FAD-dependent oxidoreductase (*Paenibacillus* sp. CC-CFT747)	0.0	96.9
CrtNa (ORF10)	WP_076265663	Phytoene desaturase family protein (*Paenibacillus* sp. FSL A5-0031)	0.0	66.8
ORF11	WJH34810	Cobalamin B12-binding domain-containing protein (*Paenibacillus* sp. CC-CFT747)	8 × 10^−122^	84.3
CrtO (ORF12)	WP_082882581	Glycosyl-4,4’-diaponeurosporenoate acyltransferase (*Pseudalkalibacillus* sp. FJAT-53715)	5 × 10^−47^	47.3
GT2 (ORF13)	WJH34812	Glycosyltransferase (*Paenibacillus* sp. CC-CFT747)	0.0	92.8
Aldedh (ORF14)	WP_099520946	Aldehyde dehydrogenase family protein (*Paenibacillus* sp. BIHB 4019)	4 × 10^−176^	55.1
ORF15	WJH34813	Phytoene desaturase family protein (*Paenibacillus* sp. CC-CFT747)	0.0	96.8
ORF16	WJH34814	Dabb family protein (*Paenibacillus* sp. CC-CFT747)	4 × 10^−63^	98.0

## Data Availability

The datasets for this study can be found in online repositories. The NCBI GenBank accession number for the 16S rRNA gene sequence of strain MBLB1776^T^ is MW130723 (https://www.ncbi.nlm.nih.gov/nuccore/MW130723, accessed on 24 October 2020). The NCBI BioProject accession number for the whole-genome sequences of strain MBLB1776^T^ is PRJNA807736 (https://www.ncbi.nlm.nih.gov/nuccore/PRJNA807736, accessed on 16 February 2022).

## Supplementary Materials

The following supporting information can be downloaded at: https://www.mdpi.com/article/10.3390/microorganisms11112719/s1, Figure S1: Negatively stained transmission electron micrograph of strain MBLB1776^T^. Strain MBLB1776^T^ was cultivated at 30 °C for three days in R2A. Bar: 2 µm; Figure S2: Thin-layer chromatograms of the polar lipids of strain MBLB1776^T^. The 1st-D developing agent, chloroform:methanol:water (65:25:4, *v*/*v*/*v*) and the 2nd-D agent, chloroform:acetic acid:methanol:water (80:15:12:4, *v*/*v*/*v*/*v*). Sulfuric acid–ethanol (1:2, *v*/*v*) solution was used for visualization of total lipids, Zinzade’s regent (5% of ethanolic molybdatophosphoric acid) for total phospholipids, ninhydrin for amino-containing lipids. The major polar lipids are DPG, PG, and PE. PL, unidentified phospholipid; APL, unidentified aminolipid; Table S1: 16S rRNA gene similarities between *Paenibacillus aurantius* MBLB1776^T^ and closely related type strains; Table S2: Description of the characteristics of novel species *Paenibacillus aurantius* MBLB1776^T^ using the API 50CH; Table S3. Differential cellular fatty acid contents of *Paenibacillus aurantius* MBLB1776^T^ and other related species. Taxa: 1, Strain MBLB1776^T^; 2. *P. cavernae* C4-5^T^; 3. *P. contaminans* CKOBP-6^T^; and 4. *P. doosanensis* CAU 1055^T^. All data are from this study under the same conditions; Table S4: Subsystem category distribution in *Paenibacillus aurantius* MBLB1776^T^ genome; Table S5: COG categories of coding proteins in *Paenibacillus aurantius* MBLB1776^T^ genome.
