# Supplementary material for: A Novel Carotenoid-Producing Bacterium, Paenibacillus aurantius sp. nov., Isolated from Korean Marine Environment
Source: Microorganisms. 2023 Nov 7;11(11):2719. doi: 10.3390/microorganisms11112719 (PMC10673419; doi:10.3390/microorganisms11112719)
Supplement: Supplementary file 1 [file microorganisms-11-02719-s001.zip › microorganisms-2678694-supplementary.pdf]

microorganisms

MDPI

Supplementary Material

A Novel Carotenoid-Producing Bacterium, *Paenibacillus aurantius* sp. nov., Isolated from Korean Marine Environment

Chi Young Hwang[1,†], Sung Man Seo[2,†], Eui-Sang Cho[1], Young-Do Nam[3], So-Lim Park[3], Seong-Il Lim[3,*], and Myung-Ji Seo[1,4,5,*]

[1] Department of Bioengineering and Nano-Bioengineering, Incheon National University, Incheon 22012, Republic of Korea; hcyoung28@inu.ac.kr (C.Y.H.); whdmltkd123@gmail.com (E.-S.C.)

[2] Advanced Geo-materials Research Department, Pohang Branch, Korea Institute of Geoscience and Mineral Resources, Pohang 37559, Republic of Korea; smseo@kigam.re.kr (S.M.S.)

[3] Personalized Diet Research Group, Korea Food Research Institute, Wanju 55365, Republic of Korea; youngdo98@kfri.re.kr (Y.-D.N.); slpark@kfri.re.kr (S.-L.P.)

[4] Division of Bioengineering, Incheon National University, Incheon 22012, Republic of Korea

[5] Research Center for Bio Materials & Process Development, Incheon National University, Incheon 22012, Republic of Korea

* Correspondence: silim@kfri.re.kr (S.-I.L.); mjseo@inu.ac.kr (M.-J.S.)

† These authors contributed equally to this work.

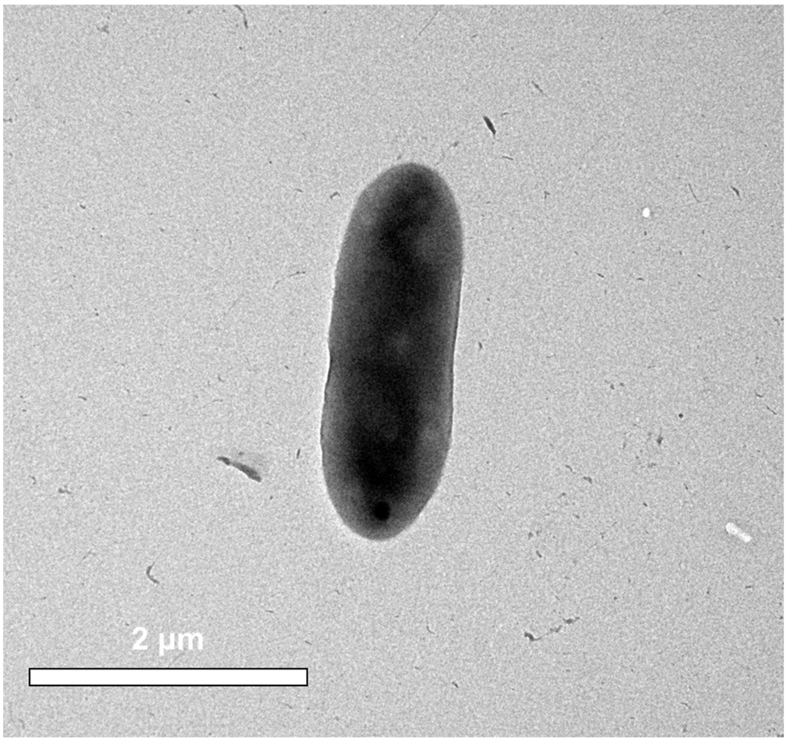

Figure S1. Negatively stained transmission electron micrograph of strain MBLB1776^T. Strain MBLB1776^T was cultivated at 30 ºC for two days in R2A. Bar: 2 µm**.**

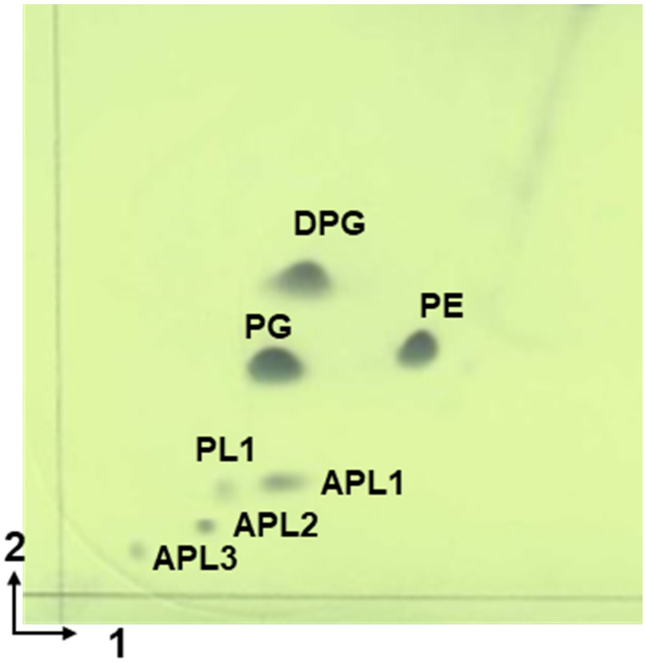

Figure S2. Thin-layer chromatograms of the polar lipids of strain MBLB1776^T. The 1st-D developing agent, chloroform:methanol:water (65:25:4, v/v/v) and the 2nd-D agent, chloroform:acetic ac-id:methanol:water (80:15:12:4, v/v/v/v). Sulfuric acid-ethanol (1:2, v/v) solution was used for visualization of total lipids, Zinzade's regent (5% of ethanolic molybdatophosphoric acid) - for total phospholipids, ninhydrin - for amino-containing lipids. The major polar lipids are DPG, PG, and PE. PL, unidentified phospholipid; APL, unidentified aminolipids;

Table S1. 16S rRNA gene similarities between *Paenibacillus aurantius* MBLB1776^T and closely related species.

Taxon	Accession no.	Similarity (%)
Paenibacillus cavernae C4-5^T	LN852381	94.83
Paenibacillus contaminans CKOBP-6^T	EF626690	94.13
Paenibacillus elgii SD17^T	AY090110	93.78
Paenibacillus chinjuensis WN9^T	AF164345	93.65
Paenibacillus piri MS74^T	KY445675	93.62
Paenibacillus esterisolvens CFH S0170^T	KP232905	93.51
Paenibacillus cookii LMG 18419^T	AJ250317	93.51
Paenibacillus puldeungensis CAU 9324^T	GU187433	93.37
Paenibacillus azoreducens AJ272249^T	AJ272249	93.32
Gorillibacterium massiliense G5^T	CBQR020000178	93.28
Paenibacillus cineris LMG 18439^T	AJ575658	93.24
Fontibacillus phaseoli BAPVE7B^T	KF583881	93.23
Paenibacillus doosanensis CAU 1055^T	JX233493	93.21

Table S2. Description of the characteristics of novel species *Paenibacillus aurantius* MBLB1776^T using the API 50CH.

**Positive**	In the API 50CH test, methyl-*β*-D-xyloside, D-galactose, D-glucose, D-fructose, D-mannose, methyl-*α*-D-glucoside, amygdalin, esculin, salicin, D-cellobiose, D-maltose, sucrose, D-trehalose, and D-raffinose are positive
**Negative**	In the API 50 CH test, glycerol, erythritol, D-arabinose, L-arabinose, D-ribose, D-xylose, L-xylose, D-adonitol, L-sorbose, L-rhamnose, dulcitol, inositol, D-mannitol, D-sorbitol, methyl-α-D-mannoside, N-acetylglucosamine, arbutin, D-lactose, D-melibiose, inulin, D-melezitose, starch, glycogen, xylitol, gentiobiose, D-turanose, D-lyxose, D-tagatose, D-fucose, L-fucose, D-arabitol, L-arabitol, gluconate, 2-ketogluconate, and 5-ketogluconate are negative.

Table S3. Differential cellular fatty acid contents of *Paenibacillus aurantius* MBLB1776^T and other related species. Taxa: 1, Strain MBLB1776^T; 2. *P. cavernae* C4-5^T; 3. *P. contaminans* CKOBP-6^T; 4. *P. doosanensis* CAU 1055^T. All data are from this study under the same condition.

Fatty acid	1	2	3	4
**Saturated**				
$C_{12:0}$	0.22	1.12	0.51	1.83
$C_{14:0}$	1.42	2.29	3.10	1.89
$C_{16:0}$	6.06	2.80	11.82	10.56
$C_{17:0}$	0.49	ND	0.74	ND
$C_{18:0}$	0.28	ND	0.77	0.83
**Unsaturated**				
$C_{15:1}$ ω5c	ND	2.30	ND	ND
$C_{16:1}$ ω7c alcohol	2.26	ND	ND	1.88
$C_{16:1}$ ω11c	3.72	ND	0.65	4.24
$C_{17:1}$ ω9c	0.21	ND	ND	ND
$C_{18:1}$ ω9c	0.85	4.61	1.87	4.40
$C_{20:1}$ ω9c	ND	ND	ND	ND
$C_{20:4}$ ω6,9,12,15c	ND	ND	0.67	0.53
**Branched-chain fatty acid**				
$C_{14:0}$ iso	4.05	4.02	2.39	3.02
$C_{15:0}$ iso	5.95	26.85	2.53	3.61
$C_{16:0}$ iso	10.10	3.45	8.89	14.28
$C_{17:0}$ iso	1.05	1.15	0.72	1.21
$C_{17:1}$ iso ω10c	0.51	ND	ND	0.56
$C_{11:0}$ anteiso	0.34	ND	ND	ND
$C_{13:0}$ anteiso	1.64	1.48	1.01	1.49
$C_{15:0}$ anteiso	56.28	43.92	58.71	42.15
$C_{17:0}$ anteiso	3.14	6.03	5.04	5.58
$C_{17:1}$ anteiso ω9c	0.19	ND	ND	0.30
**Summed feature**				
**3;** $C_{16:1}$ ω7c/$C_{16:1}$ ω6c	0.18	ND	0.57	0.57
**4;** $C_{17:1}$ iso I/$C_{17:1}$ anteiso	1.06	ND	ND	0.77
**8;** $C_{18:1}$ ω7c/$C_{18:1}$ ω6c	ND	ND	ND	0.33

Table S4. Subsystem category distribution in *Paenibacillus aurantius* MBLB1776^{T} genome

Subsystem feature	Count
Cofactors, vitamins, prosthetic groups, pigments	130
Cell wall and capsule	71
Virulence, disease and defense	59
Potassium metabolism	3
Miscellaneous	20
Membrane transport	29
Iron acquisition and metabolism	15
RNA metabolism	57
Nucleosides and nucleotides	88
Protein metabolism	200
Cell division and cell cycle	5
Motility and chemotaxis	6
Regulation and cell signaling	15
Secondary metabolism	9
DNA metabolism	83
Fatty acids, lipids, and isoprenoids	29
Nitrogen metabolism	6
Dormancy and sporulation	44
Respiration	61
Stress response	36
Metabolism of aromatic compounds	11
Amino acids and derivatives	254
Sulfur metabolism	28
Phosphorus metabolism	32
Carbohydrates	253
**Total**	**1544**

Table S5. COG categories of coding proteins in *Paenibacillus aurantius* MBLB1776^{T} genome

Code	Description	Count	%
J	Translation, ribosomal structure, and biogenesis	178	3.43
K	Transcription	445	8.58
L	Replication, recombination and repair	175	3.37
D	Cell cycle control, cell division, chromosome partitioning	35	0.67
V	Defense mechanisms	86	1.66
T	Signal transduction mechanisms	294	5.67
M	Cell wall/membrane/envelope biogenesis	228	4.39
N	Cell motility	43	0.83
U	Intracellular trafficking, secretion, and vesicular transport	40	0.77
O	Posttranslational modification, protein turnover, chaperones	105	2.02
C	Energy production and conversion	177	3.41
G	Carbohydrate transport and metabolism	637	12.28
E	Amino acid transport and metabolism	289	5.57
F	Nucleotide transport and metabolism	86	1.66
H	Coenzyme transport and metabolism	116	2.24
I	Lipid transport and metabolism	63	1.21
P	Inorganic ion transport and metabolism	231	4.45
Q	Secondary metabolites biosynthesis, transport, and catabolism	65	1.25
B	Chromatin structure and biogenesis	2	0.04
Z	Cytoskeleton	4	0.08
A	RNA processing and modification	1	0.02
S	Function unknown	1,889	36.40
**Total**		**5,189**	**100.00**
